# PET imaging of Aspergillus infection using Zirconium-89 labeled anti-β-glucan antibody fragments

**DOI:** 10.1007/s00259-024-06760-4

**Published:** 2024-05-24

**Authors:** Jianhao Lai, Swati Shah, Neysha Martinez-Orengo, Rekeya Knight, Eyob Alemu, Mitchell L. Turner, Benjamin Wang, Anna Lyndaker, Jianfeng Shi, Falguni Basuli, Dima A. Hammoud

**Affiliations:** 1 Center for Infectious Disease Imaging (CIDI), Radiology and Imaging Sciences, Clinical Center (CC), National Institutes of Health (NIH), 10 Center Drive, Room 1C368, Bethesda, MD 20892 USA; 2 Chemistry and Synthesis Center, National Heart, Lung, and Blood Institute (NHLBI), NIH, Rockville, MD USA

**Keywords:** PET imaging, Aspergillus infection, Fungal β-glucan, Antibody and fragment

## Abstract

**Purpose:**

Invasive fungal diseases, such as pulmonary aspergillosis, are common life-threatening infections in immunocompromised patients and effective treatment is often hampered by delays in timely and specific diagnosis. Fungal-specific molecular imaging ligands can provide non-invasive readouts of deep-seated fungal pathologies. In this study, the utility of antibodies and antibody fragments (Fab) targeting β-glucans in the fungal cell wall to detect Aspergillus infections was evaluated both in vitro and in preclinical mouse models.

**Methods:**

The binding characteristics of two commercially available β-glucan antibody clones and their respective antigen-binding Fabs were tested using biolayer interferometry (BLI) assays and immunofluorescence staining. In vivo binding of the Zirconium-89 labeled antibodies/Fabs to fungal pathogens was then evaluated using PET/CT imaging in mouse models of fungal infection, bacterial infection and sterile inflammation.

**Results:**

One of the evaluated antibodies (HA-βG-Ab) and its Fab (HA-βG-Fab) bound to β-glucans with high affinity (K_D_ = 0.056 & 21.5 nM respectively). Binding to the fungal cell wall was validated by immunofluorescence staining and in vitro binding assays. ImmunoPET imaging with intact antibodies however showed slow clearance and high background signal as well as nonspecific accumulation in sites of infection/inflammation. Conversely, specific binding of [^89^Zr]Zr-DFO-HA-βG-Fab to sites of fungal infection was observed when compared to the isotype control Fab and was significantly higher in fungal infection than in bacterial infection or sterile inflammation.

**Conclusions:**

[^89^Zr]Zr-DFO-HA-βG-Fab can be used to detect fungal infections in vivo. Targeting distinct components of the fungal cell wall is a viable approach to developing fungal-specific PET tracers.

**Supplementary Information:**

The online version contains supplementary material available at 10.1007/s00259-024-06760-4.

## Introduction

Invasive fungal infections (IFIs) are life-threatening diseases mainly seen in immunocompromised and immunodeficient patients [1, 2]. *Aspergillus fumigatus* (*A. fumigatus*) is the most common IFI pathogen, affecting over 250,000 patients annually [3]. The mortality rate of IFIs is exacerbated by the low sensitivity and specificity of available diagnostic tests. Conventional imaging techniques (e.g. computed tomography (CT)) are often nonspecific with radiological signatures overlapping with bacterial/viral infections, neoplasms or inflammatory processes [4]. Molecular imaging techniques such as positron emission tomography (PET), on the other hand, can potentially provide rapid, non-invasive and accurate diagnosis of fungal infection if tracers with high affinity and selectivity for fungi are developed [5].

**Fig. 1:**
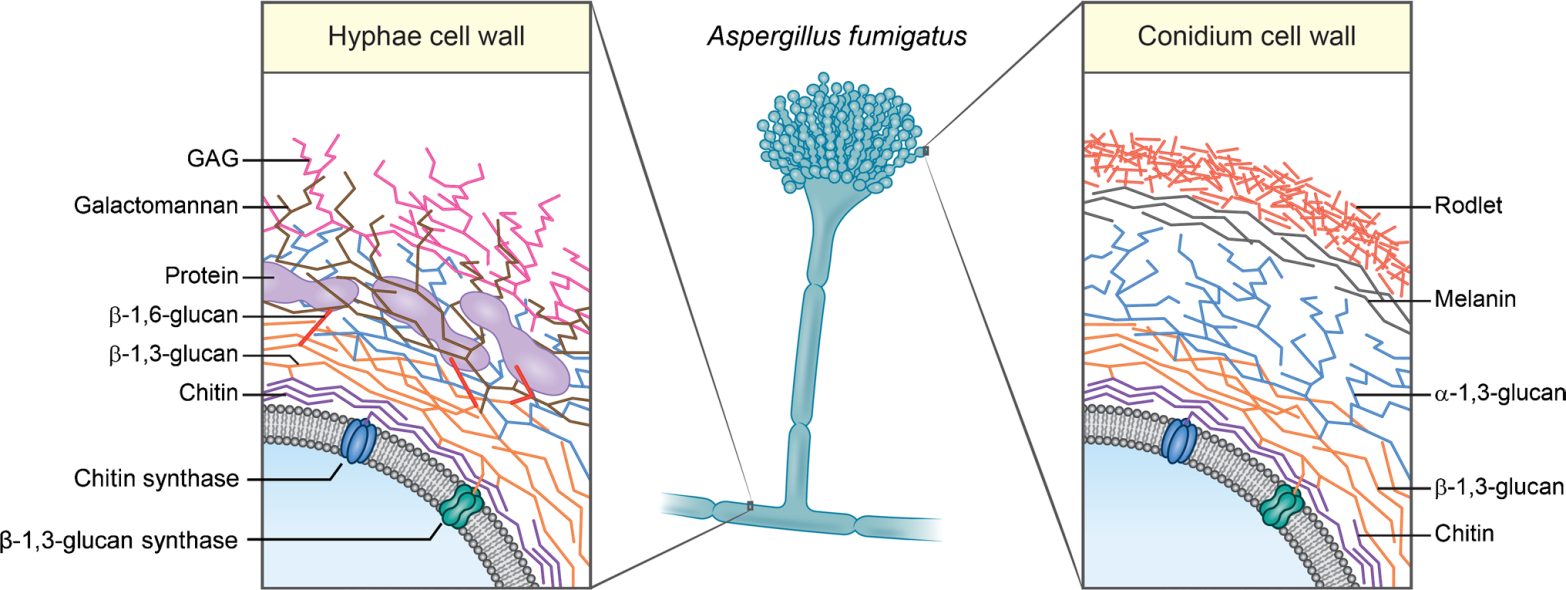
Structural organization of the cell wall of *Aspergillus fumigatus* (hyphae and conidia). GAG: Galactosaminogalactan

One potential target for fungal-specific imaging is the fungal cell wall, which encompasses multiple molecular structures that are not innately present in the human body but are often essential for fungal growth, viability and virulence (Fig. 1). Just as the unique makeup of the fungal cell wall provides ideal targets for the design of antifungal drugs [6], it could also provide targets for fungal-specific imaging. While the outer layers of the fungal cell wall are variable across fungal species, the inner cell wall in most species consists of a core of chitin and layers of branched β-(1,3) glucans [7]. β-Glucans, the most abundant cell wall polysaccharides, are known to be immunostimulatory through recognition by Dectin-1, a pattern-recognition receptor (PRR), leading to the initiation of the innate immune response [6]. Soluble β-glucans are also seen in the sera of patients with fungal infections and their detection is an important diagnostic method [8, 9].

Monoclonal antibodies (mAbs) with their superior targeting specificity have attracted great interest as potential PET imaging tracers for cancer imaging [10, 11]. Similar work in the field of fungal infection has been done successfully using JF5, an antibody targeting β-1,5-galactofuranose, a cell wall polysaccharide found in many fungal species and released into the blood [12, 13]. Radiolabeled full antibodies, however, can have multiple drawbacks including large size (150 kDa) with secondary long circulation time in blood, high radiation dose and high background signal [14]. They can also show delayed clearance from foci of infection and inflammation, most likely due to increased vascular permeability leading to interstitial extravascular leakage and retention of macromolecules, a process known as “enhanced permeability and retention (EPR) effect” [15]. Antibody fragments, on the other hand, are smaller (50 kDa for antigen-binding fragment, Fab), with shorter biological half-life (faster clearance rates), reduced immunogenicity [16], decreased potential for accumulation in the extravascular space, and maintenance of target specificity of antibodies. The main caveat however is the decreased binding affinity compared to full antibodies [17].

In this proof-of-concept study, we evaluated the binding characteristics of two commercially-available antibody clones raised against β-glucan immunogens from either lichens (*Laminaria digitate*; clone 2G8) or mushrooms (*Lentinula edodes* and *Pleurotus ostreatus*; clone B3149M) and that we radiolabeled with Zirconium-89 (^89^Zr). While both antibodies showed good affinity for their target, we observed a high degree of nonspecific accumulation of the radiolabeled versions in vivo. Subsequently, we assessed the binding characteristics of the corresponding Fabs to fungal β-glucans and compared their PET binding characteristics in mouse models of *A. fumigatus* infection, bacterial infections, sterile inflammation and tumors.

## Materials and methods

### Antibodies and antibody fragments (Fab) summary

The first clone is a mouse anti-1,3-β-glucan recombinant IgG antibody purchased from Creative Biolabs (NY, USA) (clone 2G8, Cat# MOB-0228MC) and is from here on denoted as **l**ow-**a**ffinity β-glucan antibody (LA-βG-Ab), along with corresponding murine isotype control antibody Ab (Mu-iso-Ab) (Cat# MOB-203CQ).

The second clone is a rabbit anti-β-glucan IgG antibody purchased from Biorbyt (Cambridge, UK) (clone B3149M, Cat# orb421066) and is from here on denoted as **h**igh-**a**ffinity β-glucan antibody (HA-βG-Ab). The rabbit IgG isotype control antibody (Ra-iso-Ab, Cat# MAB1050) was purchased from R&D Systems (Minneapolis, MN).

The β-glucan Fab fragments (LA-βG-Fab or HA-βG-Fab) and the isotype control Fab fragments (Mu-iso-Fab and Ra-iso-Fab) were generated by digesting the respective antibodies using Fab preparation kits (Thermo Fisher Scientific, Rockford, IL) based on the manufacturer’s instructions. Briefly, each antibody was desalted and digested by immobilized papain at 37 °C under constant stirring. Incubation time depended on the antibody’s species and concentration. The NAb Protein A Plus Spin Column (Thermo Fisher Scientific, Rockford, IL) was used to separate the Fab fragments from undigested IgG or Fc fragments. A summary of the antibodies and Fabs can be found in Table S1.

### Affinity measurements by bio‑layer interferometry (BLI) assay

The binding affinities of the antibodies and their fragments to biotinylated laminarin (a storage β-1,3-glucan of brown algae with β-1,6-linked branches, Cat# LR-BN-1, Nanocs, New York, NY), were assessed using an Octet k2 system (Satorius, Bohemia, NY). Additional details are included in supplementary data.

### Radiolabeling of antibodies and Fabs with Zirconium-89

Conjugations of *p*-isothiocyanatobenzyl-desferrioxamine (DFO-Bz-NCS) to antibodies or Fabs and ^89^Zr labeling were performed following published methods [18, 19]. Additional details are available in supplementary data.

### Saturation binding assay

To assess the affinity of [^89^Zr]Zr-DFO-HA-βG-Fab (^89^Zr-HA-βG-Fab) to β-glucans within intact fungal cell walls, saturation binding assays were performed. Since Candida and Aspergillus have a similar β-(1,3) glucan-chitin core in their cell wall [7], we chose to use *Candida albicans* (*C. albicans)* because its unicellular morphology was better suited for this experimental procedure compared to Aspergillus which tends to form long multicellular hyphae in suspension, resulting in tightly formed pellets with limited access to the antibody or fragment for binding assessment. Thus, *C. albicans* (2 × 10^6^ cells/mL) cultures were incubated at 4 °C with increasing Fab concentrations (1.6–50 nM), as described elsewhere [20]. 1 µM cold Fab fragment was used to quantify nonspecific binding. The saturation binding curves were plotted, and the affinity constants (K_D_) were determined using GraphPad Prism 8 (San Diego, CA).

### Bacterial and fungal strains

Sources of microorganisms used in this study are included in supplementary data.

### Fungal, bacterial and sterile inflammation animal models

Female CD-1 mice (6–7 weeks old, Charles River, Charleston, SC) were used for *A. fumigatus* infection and inflammation models, and female BALB/c mice (6–8 weeks old, Charles River, Charleston, SC) were used for lung cancer and bacterial myositis models. The respective mouse strains used were those which provided the most reliable and reproducible models in our hands [21]. All mice were housed in pre-sterilized filter-topped cages and given access to food and water ad libitum.

CD-1 mice were immunosuppressed by injecting cyclophosphamide intraperitoneally (IP) 4 days (150 mg/kg) and 1 day (100 mg/kg) before infection. For the pulmonary aspergillosis model, mice received 2–5 × 10^7^ *A. fumigatus* conidia via post-pharyngeal aspiration (PPA) as previously described [21]. Briefly, a 30 µL suspension of fungal conidia was pipetted onto the caudal oropharynx with the mouse kept in a vertical position and the tongue held out for at least 3 breaths. For the *A. fumigatus* myositis model, 100 µL suspensions containing either 5 × 10^7^ live or 5 × 10^7^ heat-killed *A. fumigatus* spores were injected intramuscularly (IM) into the right and left thighs of the immunosuppressed mice respectively. The radiolabeled Fabs were injected 2 days after inoculation, while the radiolabeled full antibodies were injected 5 h after inoculation.

For the bacterial myositis models, the mice were injected IM with either 1 × 10^8^ live *S. aureus* or 5 × 10^8^ live *E. coli* in the right thigh and 1 × 10^10^ heat-killed bacteria in the left thigh. The Fab tracers were injected 5 h after inoculation.

For the lung tumor model, BALB/c mice were intravenously injected with 5 × 10^5^ 4T1 (ATCC CRL-2539) tumor cells two weeks before imaging. The growth of the lung tumors was monitored using CT.

For the lung inflammation model, CD-1 mice were inoculated with polyinosinic–polycytidylic acid (poly(I:C)) (cat# tlrl-picw, Invivogen, San Diego, CA), a synthetic double-stranded RNA, using a PPA approach [21]. A 25 µL suspension containing 200 µg of poly(I:C) was administered daily for 3 days. The radiolabeled Fabs were injected 24 h after the last treatment.

For the LPS sterile myositis model, a 50 µL suspension containing 75 µg of LPS (cat# tlrl-3pelps, Invivogen, San Diego, CA) was injected intramuscularly into the left thigh of mice 24 h before injection of the tracers.

### PET/CT imaging

Small animal PET/CT imaging was performed using a nanoScan PET/CT (Mediso, Budapest, Hungary). Mice were imaged after injection of radiolabeled antibodies or Fab tracers (~ 9.25 MBq in 0.1 mL) via the tail vein. Matched isotype control antibodies/Fabs were used as negative controls. For ^89^Zr-labeled full antibodies, PET/CT imaging was performed 24, 48 and 72 h after administration. For ^89^Zr-labeled Fabs, PET/CT imaging was performed 5 and 24 h after administration. However, to achieve the best image contrast after sufficient clearance based on the half-life of antibodies (6–8 days) and Fabs (12–20 h) [22, 23], and similar to other publications [24, 25], the imaging timepoints we are showing for antibodies and Fabs were at the 72 and 24 h timepoints, respectively.

Following image reconstruction (additional details in supplementary data), volumes of interest (VOIs) were drawn over the tissues of interest based on the CT or PET images and were analyzed using MIM software (MIM Software Inc, Beachwood, OH). For myositis models only, the VOIs were drawn using PET images since the infective lesions were not clearly delineated on CT (Fig. S1). Quantifications are presented as mean standardized uptake values (SUVmean). After imaging, the mice were euthanized and their thigh muscles, blood, and major organs/tissues were harvested. Radioactivity in tissues was measured using a gamma counter (PerkinElmer, Chicago, IL), and the results are presented as %ID/g for ex vivo biodistribution.

### Grocott's methenamine silver (GMS) and immunofluorescence staining

Details are included in supplementary data.

### Statistical analyses

GraphPad Prism 8 software (GraphPad Software, San Diego, CA) was used for data analyses. Statistical analysis was done using 2-way ANOVA or unpaired Student t-test based on the experimental design. A P value < 0.05 was considered statistically significant. Quantitative data are expressed as mean ± standard deviation (SD).

## Results

### Radiolabeling with Zirconium-89

The radiochemical purities of ^89^Zr-labeled antibodies and Fabs, as determined by SE-HPLC, were ≥ 97% (Fig. S2). In vitro stabilities of representative conjugates (^89^Zr-HA-βG-Ab and ^89^Zr-HA-βG-Fab) were tested in mouse serum at 37 °C (Fig. S3). The SE-HPLC chromatogram indicated slight decomposition of the radiolabeled conjugates (78% intact for ^89^Zr-HA-βG-Ab at 120 h and 76% intact for ^89^Zr-HA-βG-Fab at 48 h). Additional details are included in supplementary data.

### Assessment of the binding characteristics of β-glucan antibodies and Fab fragments

**Fig. 2:**
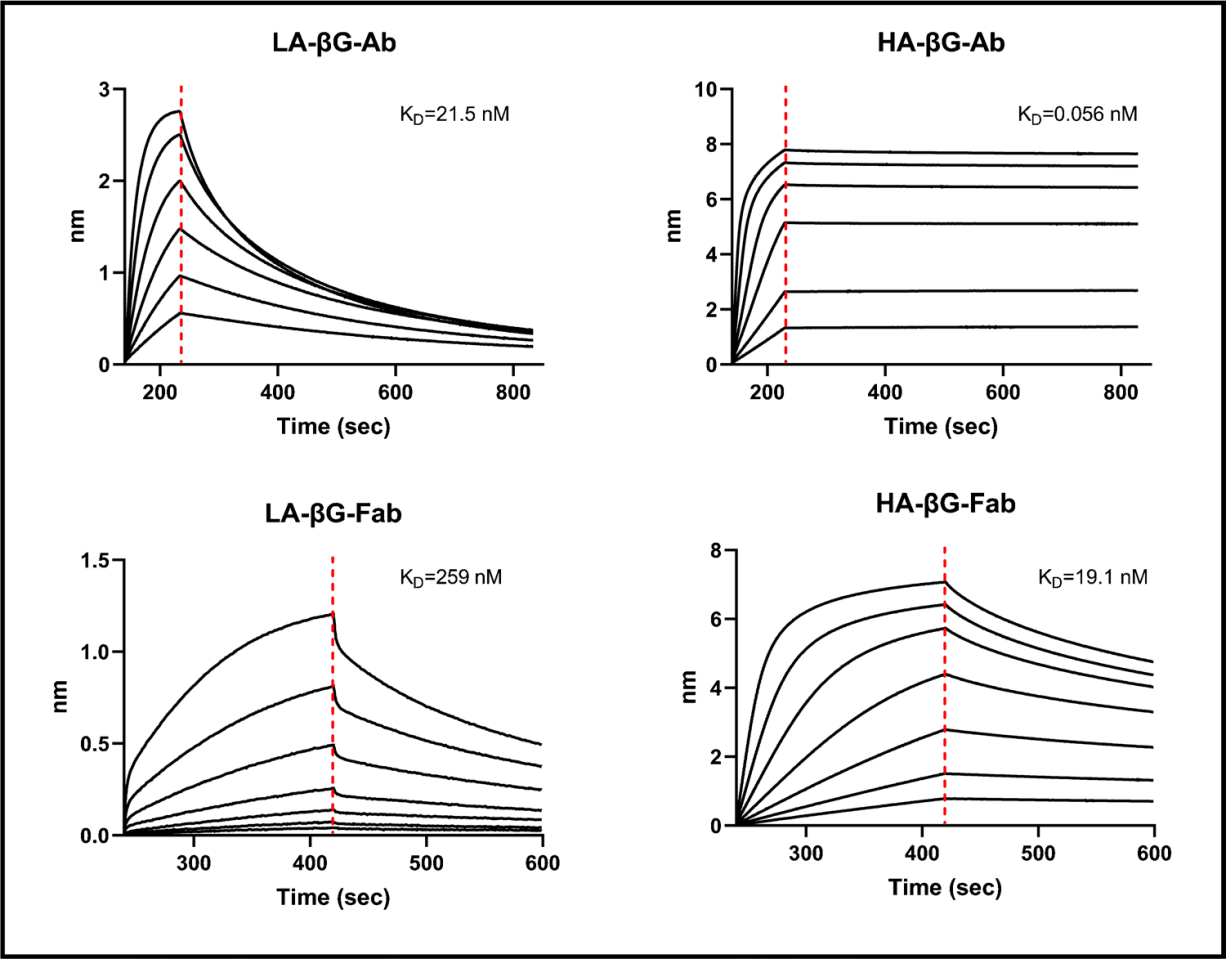
Binding affinity of anti-β-glucan antibodies or fragments (Fab) to biotinylated laminarin were determined by biolayer interferometry assay using the octet system. K_D_ = Dissociation constant

The affinity of HA-βG-Ab (K_D_ = 0.056 nM) was much higher than LA-βG-Ab (K_D_ = 21.5 nM). The K_D_ values of HA-βG-Fab and LA-βG-Fab were 19.1 nM and 259 nM, respectively (Fig. 2). As expected, the Fabs binding affinities were ~ tenfold lower than the full antibodies [26].

To ensure that the addition of the chelator for radiolabeling would not affect binding of the Fabs, a BLI assay with DFO-conjugated HA-βG-Fab (cold) was performed and K_D_ of 22.4 nM was obtained (Fig. S4A). Saturation binding assays using ^89^Zr-HA-βG-Fab in *C. albicans* cultures showed a K_D_ value of 19.6 nM, in the same range as BLI (Fig. S4B).

**Fig. 3:**
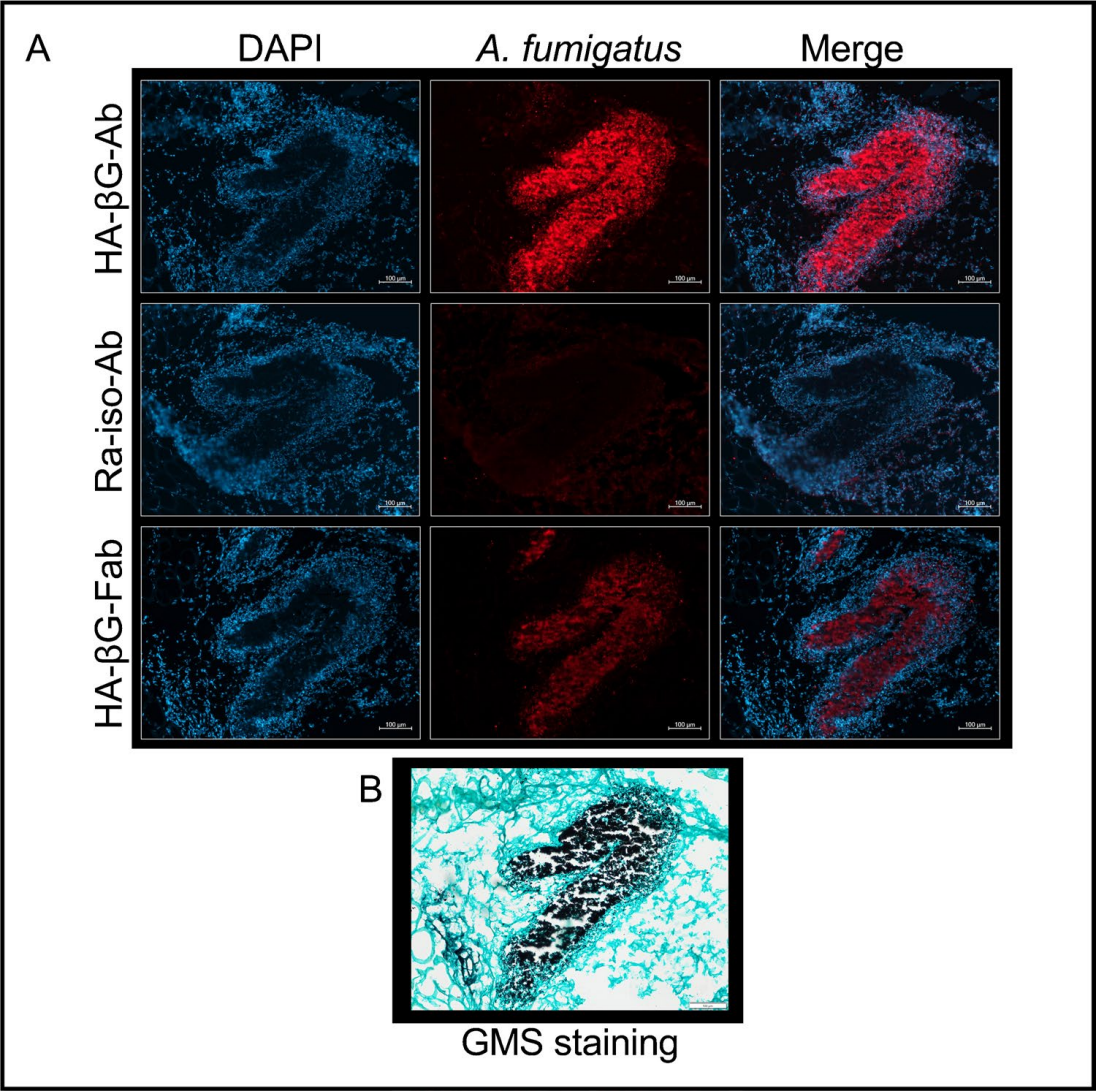
Validation of HA-βG-Ab and HA-βG-Fab binding to *A. fumigatus* by immunofluorescence microscopy. **A** Immunofluorescence staining of β-glucan of muscle tissues from *A. fumigatus* myositis model, using primary antibodies / Fabs and Alexa Fluor 647 (Red) labeled secondary antibody and counterstained with DAPI (blue). All images were acquired under the same conditions and displayed at the same scale. Scale bar: 100 mm. **B** Corresponding GMS staining confirms fungal infection

Finally, immunofluorescence staining of thigh muscle tissues from *A. fumigatus* myositis mice showed positive staining only in sections exposed to either HA-βG-Ab or HA-βG-Fab but not the isotype control antibody (Fig. 3). Similar staining patterns were observed with LA-βG-Ab and LA-βG-Fab, albeit with a lower signal intensity compared to the HA versions (Fig. S5). GMS staining performed on either the same slice or an adjacent one confirmed the location of *A. fumigatus* infection.

### Comparing in vivo target specificity of full antibodies in myositis models

#### Low-affinity antibodies and corresponding isotype controls

**Fig. 4:**
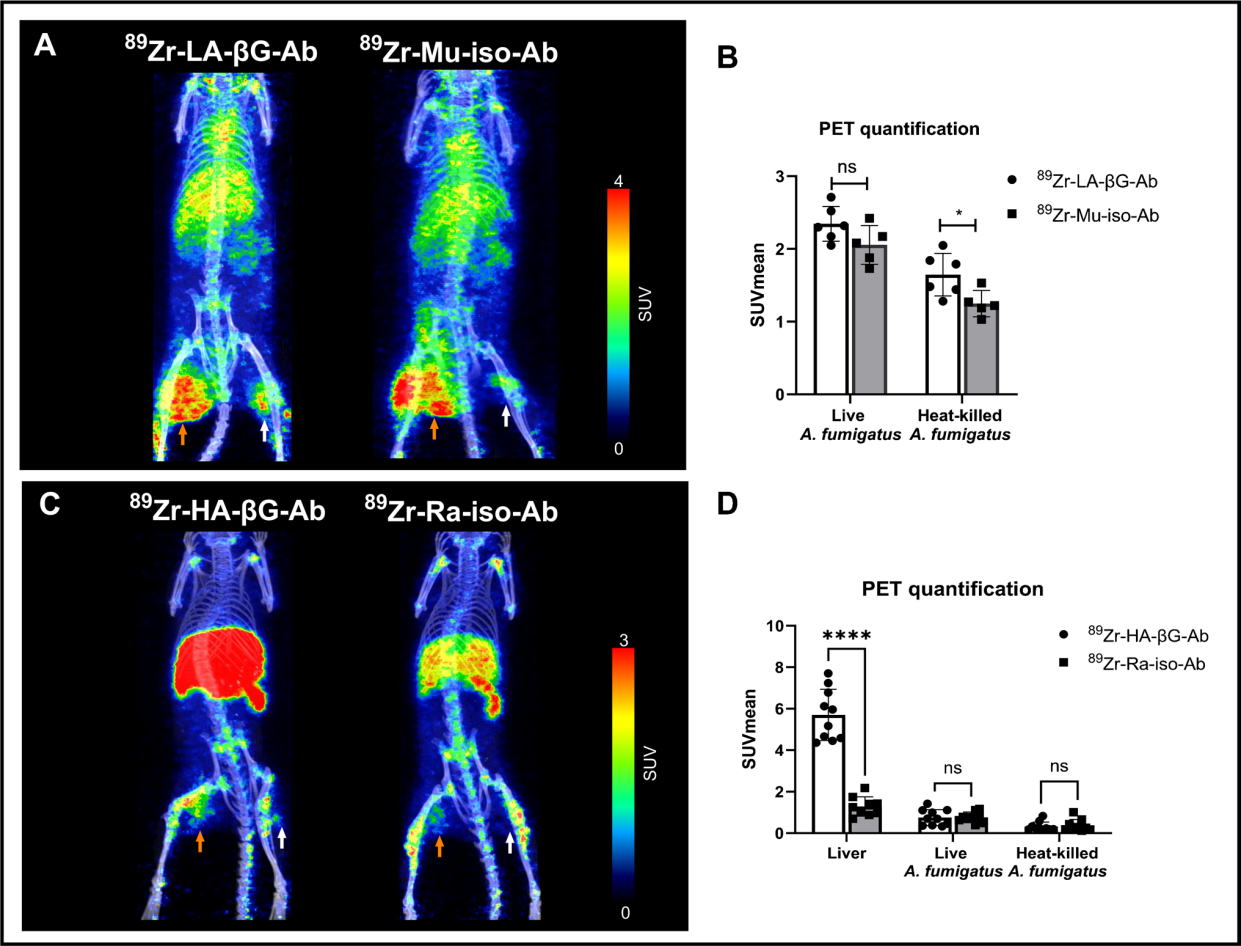
Static PET/CT imaging of mice with *A. fumigatus* myositis infections using ^89^Zr labeled full antibodies and isotype controls. **A** Maximum intensity projections (MIPs) of representative PET/CT images using ^89^Zr-LA-βG-Ab and ^89^Zr-Mu-iso-Ab and (**B**) quantified binding in the live (orange arrows) and heat-killed (HK; white arrows) injection sites at 72 h post-tracer injection. There is no significant difference between the binding of ^89^Zr-LA-βG-Ab (*n* = 6) and ^89^Zr-Mu-iso-Ab (*n* = 5) in the live *A. fumigatus* infected thigh (*P* = 0.09). However, there was slightly higher binding in heat-killed *A. fumigatus* (*, *P* < 0.05). **C** MIPs of representative PET/CT images at 72 h post injection of ^89^Zr-HA-βG-Ab and.^89^Zr-Ra-iso-Ab and (**D**) PET quantification of radioactivity in the liver, live *A. fumigatus* (orange arrows), and heat-killed *A. fumigatus* (white arrows) injection sites (*n* = 6–9). Unpaired t-test was used for statistical analysis. ****, *P* < 0.0001

In the case of ^89^Zr-LA-βG-Ab (K_D_ = 21.5 nM), higher radioactivity retention was observed in live *A. fumigatus* thigh infection (SUVmean = 2.34 ± 0.24), compared to the heat-killed side (SUVmean = 1.65 ± 0.29), 72 h after injection of the labeled antibodies. However, no significant differences in binding were found when comparing the binding of ^89^Zr-LA-βG-Ab to its isotype control ^89^Zr-Mu-iso-Ab in live infection (Fig. 4). On the heat-killed side, however, there was slightly higher binding of ^89^Zr-LA-βG-Ab compared to isotype control (Fig. 4).

#### High-affinity antibodies and corresponding isotype controls

Next, we evaluated the binding of high affinity β-glucan antibody (^89^Zr-HA-βG-Ab; K_D_ = 0.056 nM) and its corresponding isotype control (^89^Zr-Ra-iso-Ab) in *A. fumigatus* myositis mice. While this clone has much higher affinity to β-glucans, most of the ^89^Zr-HA-βG-Ab accumulated in the liver instead, likely because of aggregation associated with free β-glucans in the blood [8] with secondary shuttling of the aggregates to the liver. On the other hand, when the ^89^Zr-Ra-iso-Ab was injected, the liver binding was within expected limits. Looking specifically at the infected thighs, the SUVmean values for the ^89^Zr-Ra-iso-Ab in the thighs (0.76 ± 0.26) were similar to those of ^89^Zr-HA-βG-Ab (0.76 ± 0.38) in both the live and heat-killed sites (Fig. 4D).

### Comparing the in vivo target specificity of Fabs in myositis models

To evaluate if the removal of the Fc region and size reduction would mitigate non-specific ligand accumulation in infected mice, PET imaging with ^89^Zr-labeled LA-βG-Fab and HA-βG-Fab was performed at the 24-h timepoint along with the corresponding isotype controls.

#### Low-affinity β-glucan Fab and corresponding isotype control

First, the in vivo biodistribution of ^89^Zr-LA-βG-Fab was compared to its isotype control ^89^Zr-Mu-iso-Fab. The ligand was mostly excreted renally, as expected [27] and the background radioactivity levels were lower than seen with the intact antibodies. However, the signal retention of ^89^Zr-LA-βG-Fab and ^89^Zr-Mu-iso-Fab in *A. fumigatus* inoculation sites was comparable. Nonetheless, more binding was seen with live infection compared to the heat-killed sites with both Fabs, likely due to higher inflammation and secondary increased vascular permeability (strong EPR effect) [15] (Fig. S6).

#### High-affinity β-glucan Fab and corresponding isotype control

**Fig. 5:**
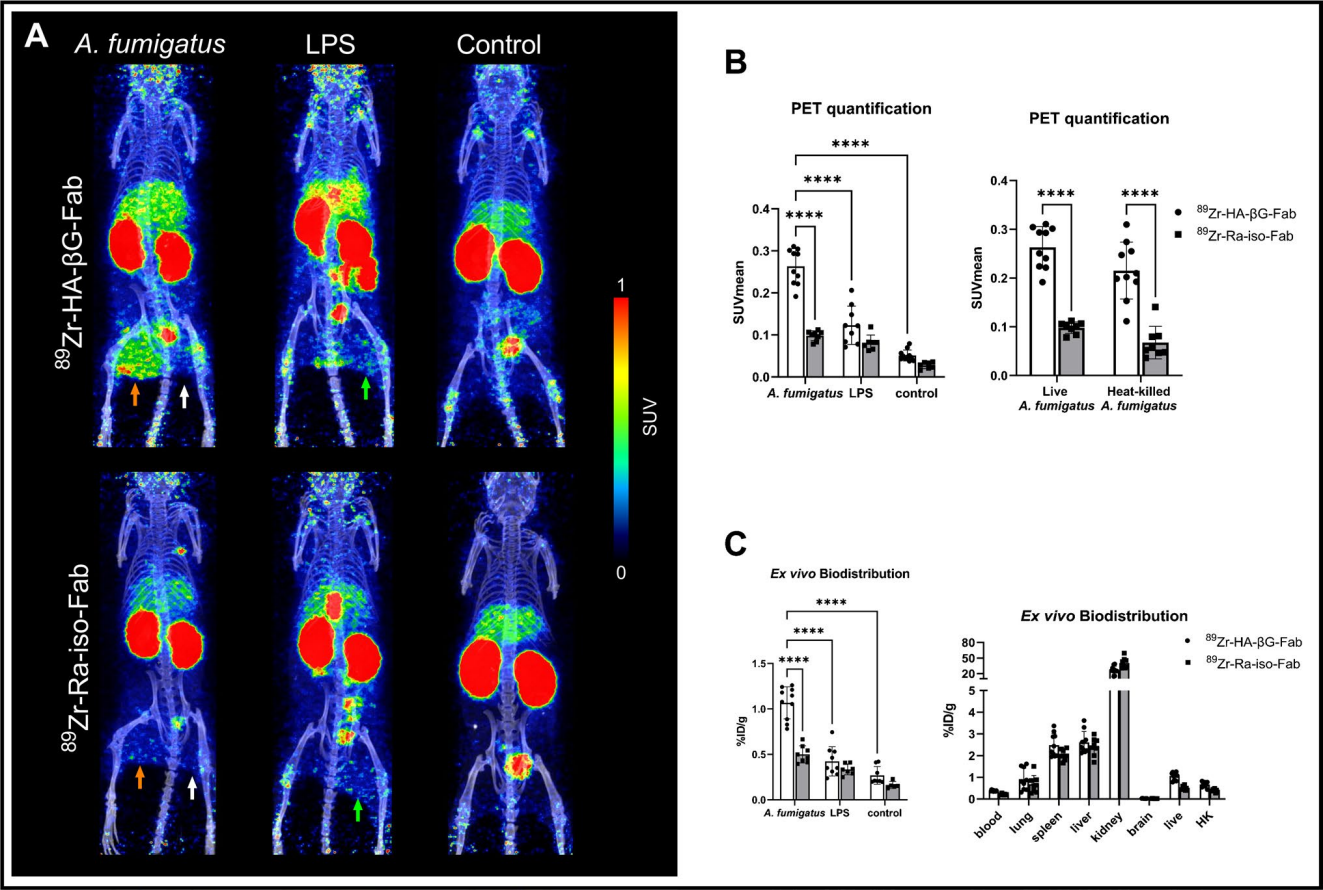
Static PET/CT imaging with ^89^Zr-HA-βG-Fab and ^89^Zr-Ra-iso-Fab in myositis models. **A** Maximum intensity projections (MIPs) of representative PET/CT images. Live *A. fumigatus* (orange arrows), heat-killed *A. fumigatus* (white arrows) and LPS (green arrows) injection sites. **B** PET quantification (*n* = 7–11) of binding in the live or heat-killed *A. fumigatus*, LPS injection sites and normal muscle in control animals at 24 h post-tracer injection. **C** Ex vivo biodistribution (*n* = 5–10) of ^89^Zr-HA-βG-Fab and ^89^Zr-Ra-iso-Fab in the live *A. fumigatus*, LPS injection sites and normal muscle or in all tissues of *A. fumigatus* myositis models after PET imaging. Two-way ANOVA with multiple comparisons was used for statistical analysis. ****, *P* < 0.0001

On the other hand, ^89^Zr-HA-βG-Fab showed significantly higher binding than ^89^Zr-Ra-iso-Fab in both live (SUVmean = 0.26 ± 0.04 vs. 0.10 ± 0.01) and heat-killed (SUVmean = 0.22 ± 0.06 vs. 0.07 ± 0.03) *A. fumigatus* infection sites (Fig. 5A, B). Representative axial view PET/CT images show the VOIs on the live and heat-killed sites (Fig. S1). GMS staining confirmed the fungal burden (Fig. S7). We concluded that the differences in binding between the two anti-β-glucan Fabs can be attributed to the lower binding affinity of ^89^Zr-LA-βG-Fab compared to ^89^Zr-HA-βG-Fab (K_D_ = 259 vs 22.4 nM).

The specificity of ^89^Zr-HA-βG-Fab was further evaluated using an LPS sterile inflammation myositis model. The binding of ^89^Zr-HA-βG-Fab in *A. fumigatus* infection was significantly higher than in LPS or control mice (SUVmean = 0.26 ± 0.04 vs. 0.12 ± 0.04 and 0.05 ± 0.01). Ex-vivo biodistribution results were consistent with the imaging results (Fig. 5). In summary, we found faster clearance and lower background signal with ^89^Zr-HA-βG-Fab compared to full antibody with binding in foci of *A. fumigatus* infection but not in sterile inflammation.

### Comparing ^89^Zr-HA-βG-Fab binding in pulmonary aspergillosis, sterile inflammation and tumor models

**Fig. 6:**
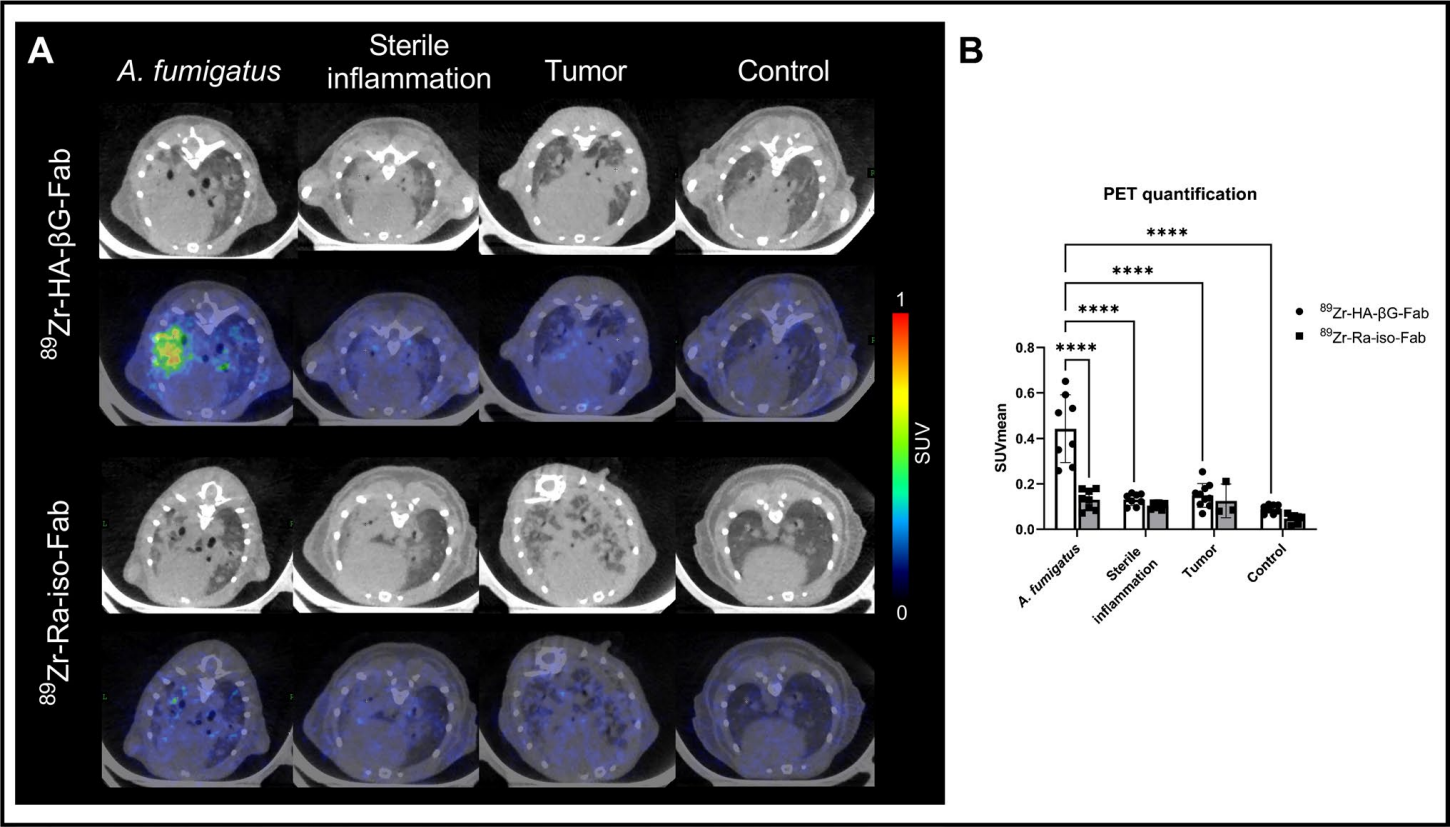
Static PET/CT imaging of *A. fumigatus* lung infection and inflammation models with ^89^Zr-HA-βG-Fab and ^89^Zr-Ra-iso-Fab. **A** Representative axial images of lung CT and PET/CT fusion are shown from *A. fumigatus* pulmonary infection (column 1), poly (I:C)-induced sterile lung inflammation (column 2), lung tumors (column 3) and healthy controls (column 4), 24 h after tracer injection. **B** PET quantification of binding in the lung consolidations (*n* = 7–10, except for tumor model injected with.^89^Zr-Ra-iso-Fab: *n* = 3). Two-way ANOVA with multiple comparisons was used for statistical analysis. ****, *P* < 0.0001

Since ^89^Zr-HA-βG-Fab showed promising results with *A. fumigatus* myositis infections, it was further evaluated in a clinically-relevant pulmonary aspergillosis model. Higher radioactivity retention of ^89^Zr-HA-βG-Fab was seen in lungs of infected mice at 24 h after tracer injection compared to controls and ^89^Zr-Ra-iso-Fab (SUVmean = 0.44 ± 0.15 vs. 0.08 ± 0.02 vs. 0.12 ± 0.04 respectively) (Fig. 6). The binding of ^89^Zr-HA-βG-Fab corresponded to regions of lung opacification on CT scans, with no binding in normal lungs. Representative axial and coronal CT images show lung infiltrates/consolidations in the *A. fumigatus* pneumonia compared to healthy mice (Fig. S8). Almost complete clearance of the ^89^Zr-Ra-iso-Fab was noted in the infected lungs at the same timepoint (Fig. 6). GMS staining confirmed pulmonary fungal infection (Fig. S7).

The binding of ^89^Zr-HA-βG-Fab in *A. fumigatus* infected lungs was also significantly higher than in lung sterile inflammation and tumor models (Fig. 6).

### Comparing ^89^Zr-HA-βG-Fab binding in *A. fumigatus* and bacterial myositis models

**Fig. 7:**
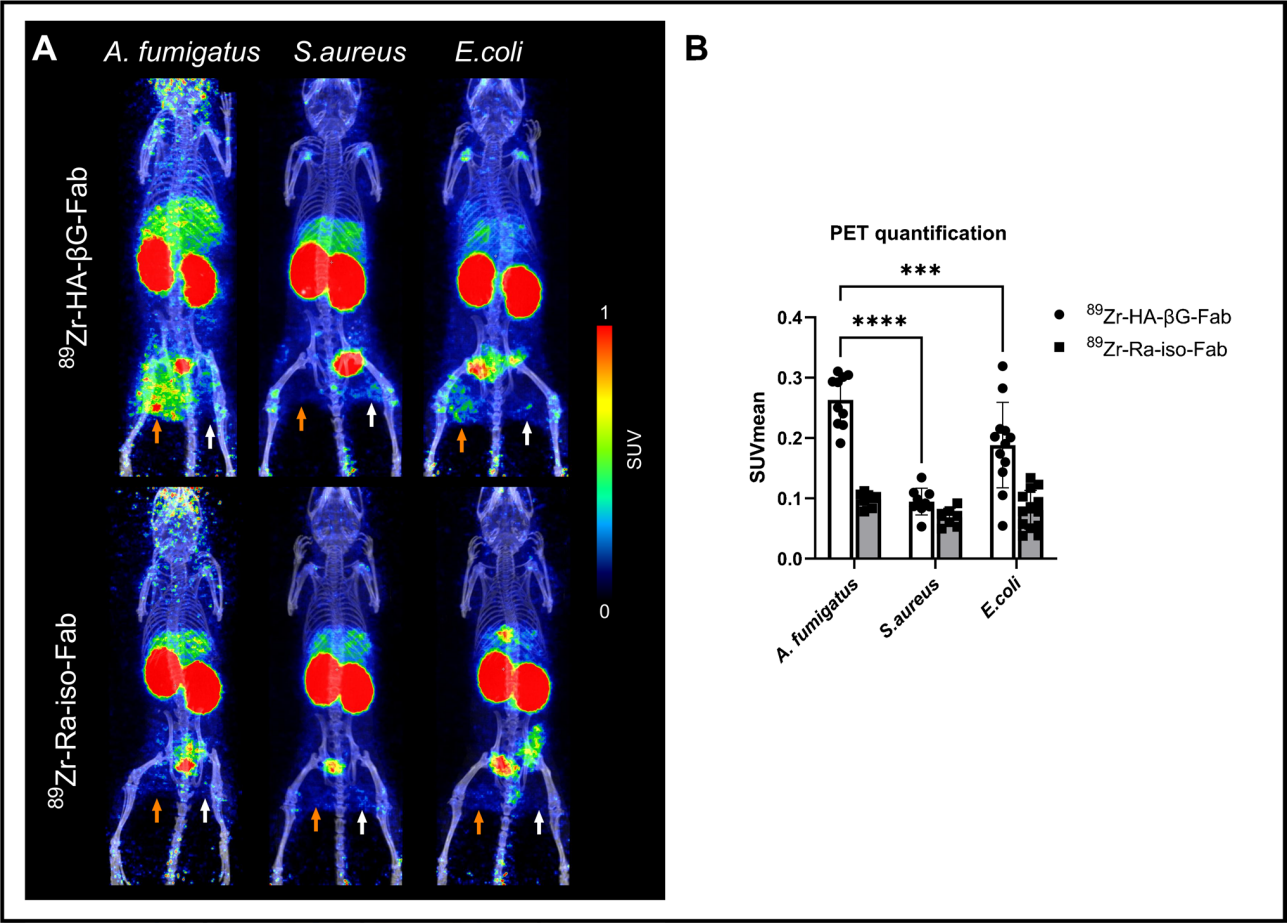
Static PET/CT imaging with ^89^Zr-HA-βG-Fab and ^89^Zr-Ra-iso-Fab of mice with *A. fumigatus* and bacterial myositis. **A** Maximum intensity projections (MIPs) of representative PET/CT images of myositis infections with either live (orange arrows) or heat-killed (white arrows) *A. fumigatus*, *S. aureus*, and *E. coli*, 24 h after tracer injection and (**B**) PET quantification of binding in live *A. fumigatus*, *S. aureus*, and *E. coli* infection sites (*n* = 7–13). Two-way ANOVA with multiple comparisons was used for statistical analysis. ***, *P* < 0.001, ****, *P* < 0.0001

Bacterial myositis mice had the same degree of swelling and lameness as those with *A. fumigatus* myositis. ^89^Zr-HA-βG-Fab binding was higher in *A. fumigatus* compared to *S. aureus* live infected thighs (SUVmean = 0.26 ± 0.04 vs. 0.09 ± 0.02) and heat-killed inoculated thighs (SUVmean = 0.22 ± 0.06 vs. 0.12 ± 0.03). There was however mild retention of radioactivity of ^89^Zr-HA-βG-Fab in the live *E. coli* inoculation site, which still remained lower than with live *A. fumigatus* infection foci (SUVmean = 0.19 ± 0.07 vs. 0.26 ± 0.04) (Fig. 7). ^89^Zr-Ra-iso-Fab showed no retention of signal in any of the models.

## Discussion

In recent years, multiple radioligands have been developed and tested as potential fungal-specific tracers, including radiolabeled peptides, antifungal drugs, siderophores, fungal-specific antibodies and sugars [5]. Among those, radiolabeled antibodies can be developed with high affinity and specificity for their target. One example is the successful development of radiolabeled antibodies against fungal β-1,5-galactofuranose [12, 13]. In a similar fashion, our study focused on imaging fungal infections by using radiolabeled antibodies and antibody fragments against fungal-specific cell wall components, many of which are not naturally present in mammalian cells. Among those are β-1,3 glucans, which in the fungal wall are characteristically associated with β-1–6 glucan side chains and are well-conserved among most fungal species [28]. Here, we used two commercial IgG antibodies targeting β-glucans with different binding affinities to laminarin, a storage brown algae glucan composed of β-1,3 glucans linked by β-1,6 glucan sidechains, which is similar to β-glucans’ arrangement in the fungal cell wall. When we labeled the two antibodies as well as their corresponding Fabs with ^89^Zr in different models of infection and inflammation, only the high-affinity Fab was able to provide adequate specific visualization of *A. fumigatus* infections foci.

During fungal infection, the host undergoes a series of vascular and cellular changes, including vasodilation and increased vascular permeability which makes it possible to detect the infectious site using nonspecific IgGs (EPR effect) [15] and their use as radiolabeled ligands has been demonstrated in various infections including *A. fumigatus*, *S. aureus* or *E. coli* infection in previous studies [29–31]. However, the detected signal in those situations is mainly due to increased vascular permeability (EPR effect) which is non-specific. This is consistent with our results using full antibodies (β-glucan-specific and IgG isotypes), all of which leaked/accumulated at sites of live, and to a lesser extent, heat-killed infections with high residual signal still seen 72 h post-injection (Fig. 4). We believe there must have been specific binding of the β-glucan-specific antibodies to *A. fumigatus* fungal cell wall, however this was obscured by nonspecific extravasation and retention of all radiolabeled antibodies in the extravascular space of the infected/inflamed regions (EPR effect [15]). Another reason for poor clearance of antibodies from the inflamed/infected region is likely the interaction between the Fc regions and Fcγ receptors, abundantly present in activated immune cells within the infectious/inflammatory milieu [32]. Conversely, radiolabeled Fabs had less accumulation in and faster clearance from the focal inflammatory sites likely due to the smaller size and lack of Fc fragment.

In addition to EPR effect problem confounding specificity, PET/CT imaging with the full anti-β-glucan antibody (^89^Zr-HA-βG-Ab) showed remarkably high radioactivity in the liver of *A. fumigatus* infected mice (Fig. 4), unlike its isotype. This is likely due to soluble β-glucans in the blood that sequester the antibody as an “antigen sink” [33], with secondary hepatic clearance of the aggregated immune complexes. This results in lower amounts of labeled antibody available to bind to the infectious focus. The ^89^Zr-HA-βG-Fab, on the other hand, did not have these drawbacks (Fig. 5A) since the hepatic clearance of immune complexes is Fc-receptor dependent [34, 35].

One question that could be raised, concerns the ability of the radiolabeled antibodies and Fabs to reach their target, β-glucans, in the fungal cell wall. Fungal β-glucans are conserved across different fungal species and are thus recognized by human innate immune system as pathogen-associated molecular patterns with secondary initiation of anti-fungal immunity [28]. They are, however, often concealed by a rodlet layer of hydrophobic proteins [36] that allow pathogens to escape immune recognition and could potentially make them inaccessible to imaging tracers. However, Torosantucci et.al. found that the 2G8 antibody (which we used as an example of low affinity β-glucan antibody) could inhibit the growth of *C. albicans *in vitro [37], suggesting that β-glucans are indeed accessible to antibodies. It appears that during germination and fast growth, the outer cell wall layers, including the rodlet and melanin layers, get degraded/disorganized, leading to the exposure of inner polysaccharide layers containing β-glucans [38, 39]. Also, under different environmental conditions, such as low glucose concentrations or hypoxia, the organization of the cell wall can change, and the β-glucan layer may emerge on the surface [28]. All this is also consistent with our immunofluorescence results showed that the anti-β-glucan antibodies specifically bound to *A. fumigatus* in infected muscle tissues (Fig. 3). This opens the door to targeting other deeply-seated fungal cell components (e.g. chitin) for imaging.

Using radiolabeled ^89^Zr-HA-βG-Fab, we were able to clearly distinguish fungal infections from sterile inflammation, malignancy and *S. aureus* infection. However, some binding in the *E. coli* infected mice was observed (Fig. 7), though it was lower than that of *A. fumigatus* infection. One possibility is that the HA-βG-Fab cross-reacts with the β-glucans found in *E. coli* even though their glucan organization is different from that of the fungal cell wall. In various gram-negative bacteria, osmoregulated periplasmic glucans which maintain the osmotic pressure inside the cell, are mostly composed of a β-1,2-glucan backbone, branched by one glucose unit attached by β-1,6 linkages [40, 41]. Recently, β-1,3 glucans were also reported to be present in some bacteria, even though to a lesser extent [42].

An additional benefit of using Fabs besides having better pharmacokinetic profiles than full antibodies, thus achieving a high target to background ratio in a shorter period of time, is approximately 90% reduction in immunogenicity compared to antibodies (through removal of the Fc region) with lower liver and bone marrow radioactive signal [14]. Fabs, however, are still limited by decreased affinity compared to the corresponding antibodies [43]. Starting with a very high affinity antibody is thus necessary to provide adequate binding of the corresponding Fab to the fungal cell wall while maintaining lower non-specific binding than intact antibodies. Fab fragments are also subject to nonspecific extravasation and extravascular retention due to EPR effects however the lack of the Fc region likely reduces non-specific binding in the extravascular space and allows faster clearance compared to full antibodies. An alternative to using Fabs would be to specially engineer the Fc region to eliminate antibody binding to Fcγ receptors and potentially reducing the nonspecific binding. Additional changes to the amino acid sequences for humanization would be needed prior to translation [14]. This has already been done successfully by other groups [12, 13]. The latter approach would still be hindered by long circulating times of radiolabeled antibodies and need for delayed imaging. Ultimately, the final choice between humanized full antibodies and antibody fragments/other engineered antibody constructs should be based on the pre-existing characteristics of the antibody, antigen and targeted pathology.

Our study has limitations. We know that our radioligands cross-react with β-glucans from *C. albicans* based on the saturation binding studies and while we assume they should bind to β-glucans from all fungal species considering the well-conserved fungal cell wall architecture [7], we have not yet tested binding with other fungi including other *A. fumigatus* strains. Those experiments are planned for future iterations of this project. Another limitation of ^89^Zr-HA-βG-Fab is the high retention in the spleen, liver and kidneys at 24 h, which is a known issue with radiolabeled antibody fragments, variants and nanobodies [44]. In a clinical setting, the Fab biodistribution/clearance however should not be an issue when assessing invasive pulmonary aspergillosis (one of the most common IFIs) considering the minimal background signal in normal lungs and heart by 24 h. This might however be an issue when assessing abdominal infectious foci.

## Conclusion

We have demonstrated that a ^89^Zr labeled antibody fragment targeting conserved fungal cell wall β-glucans can specifically detect *A. fumigatus* infections in animal models. This approach along with targeting other unique fungal cell wall components with radiolabeled antibody fragments, other specific recombinant/engineered antibodies or nanobodies are thus feasible and will likely improve the accuracy of diagnosis of invasive fungal infections. Finally, the adaptation of other engineered antibody fragments such as F(ab’)2 (which has two antigen-binding sites) targeted against fungal β-glucans as well as targeting other fungal specific wall components, such as chitin or β-1,6-glucans, is warranted.

### Supplementary Information

Below is the link to the electronic supplementary material.

Supplementary file1 (PDF 1357 KB)

## Data Availability

The datasets generated and/or analyzed during the current study are available from the corresponding author upon reasonable request.
