# Supplementary material for: PET imaging of Aspergillus infection using Zirconium-89 labeled anti-β-glucan antibody fragments
Source: Eur J Nucl Med Mol Imaging. 2024 May 24;51(11):3223–34. doi: 10.1007/s00259-024-06760-4 (PMC11368974; doi:10.1007/s00259-024-06760-4)
Supplement: Supplementary file 1 — Supplementary file1 (PDF 1357 KB) [file 259_2024_6760_MOESM1_ESM.pdf]

PET imaging of Aspergillus infection using Zirconium-89 labeled anti-β-glucan antibody fragments

Jianhao Lai[1†], Swati Shah[1†], Neysha Martinez-Orengo[1], Rekeya Knight[1], Eyob Alemu[1], Mitchell Turner[1], Benjamin Wang[1], Anna Lyndaker[1], Jianfeng Shi[2], Falguni Basuli[2], Dima A. Hammoud[1*]

Supplementary data

Materials and Methods

Affinity measurements by bio-layer interferometry (BLI) assay

1X Phosphate buffered saline (PBS) was used as the buffer system for this assay. Streptavidin (SA) biosensors (Cat# Nos.18-5021 Satorius) were blocked with 1% BSA, after which they were loaded with 0.5 µg/mL biotinylated laminarin (β-1,3 and 1,6 glucans). These sensors were then exposed to either a full Ab or Fab of different concentrations (0~250 nM) and finally dipped in PBS for the dissociation phase. Data analysis and curve fitting were performed using Octet Analysis Studio 13.0 (Satorius). The bio-layer interferometry (BLI) measurements for each antibody or fragment were performed at least twice.

Radiolabeling of antibody and fragments with Zirconium-89

The p- isothiocyanatobenzyl - desferrioxamine (DFO-Bz-NCS) was purchased from Macrocyclics, Inc. (Plano, TX, USA). Sodium acetate and HEPES buffer were obtained from Thermo Fisher Scientific (Waltham, MA, USA). MilliporeSigma (St. Louis, MO, USA) and used without further purification. PD-10 desalting columns were obtained from GE Healthcare Biosciences (Pittsburgh, PA, USA). Zirconium-89 oxalate was obtained from 3D Imaging (Little Rock, Arkansas, USA). Analytical high-performance liquid chromatography (HPLC) analyses were performed on an Agilent 1200 Series instrument equipped with a multi-wavelength UV detector connected in series with a Bioscan flow count radio detector. The size-exclusion column (SE, 4.6 mm ID x 30 cm, 4µm), TSKgel SuperSW3000, was obtained from Tosoh Bioscience LLC. (King of Prussia, PA, USA). HPLC condition: eluent, 0.1 M sodium phosphate, 0.1 M sodium sulfate, 0.05% sodium azide, 10% isopropyl alcohol (pH 6.8), flow rate: 0.3 mL/min. BCA Protein Assay Kit (Thermo Fisher Scientific) with bovine gamma globulin standard was used to determine the conjugate concentrations.

Full antibodies or fragments were buffer-exchanged into 0.1 M $NaHCO_3$ (containing 0.9% NaCl, pH 8.9), concentrated to 3-5 mg/mL and mixed with 5-fold molar excess of DFO-Bz-NCS (5 mg/mL in DMSO). The mixture was gently rocked at 37 $^{\circ}$C for 75 min before stopping the conjugation reactions by the addition of 1M Tris (to a final concentration of 12-15 mM). The DFO conjugates were purified twice by two PD-10 columns using 0.5 M HEPES buffer (pH 7.1 - 7.3) as an eluant. The concentrations of DFO-antibodies/fragments were determined by using bicinchoninic acid assay (BCA). The purities of the conjugates were determined by high performance liquid chromatography (HPLC) using a size exclusion column (SE-HPLC).

A stock solution of zirconium-89 oxalate was diluted with 300 µL of HEPES buffer (0.5 M, pH 7.1 -7.3). From this stock solution, ~150 MBq of zirconium-89 was used per radiolabeling reaction. The aliquot of zirconium-89 oxalate (~150 MBq, 110 uL) was further diluted with HEPES buffer (0.5 M, 770 µL, pH 7.1 -7.3). 2,5-Dihydroxybenzoic acid (20 µL, ~5 mg/mL in water, pH adjusted to 7 with 2M Na_2CO_3 solution) was added followed by a solution of DFO-conjugates (0.1 - 0.2 mg, 100 µL). The reaction mixture was incubated for 1 h at room temperature and challenged with DTPA (5 µL, 0.1 M, pH 7) for an additional 10 min. The radiolabeled conjugates were purified by PD-10 column using 0.9% NaCl (pH 7).

To test the stability of ^{89}Zr-HA-βG-Ab and ^{89}Zr-HA-βG-Fab, 500 µL of mouse serum was added to the solution of conjugates (~500 µCi in 500 µL of saline, pH 7.0). The solution was kept at 37 °C for up to 7 days for antibody and 2 days for fragment. The radiochemical stability was determined by directly injecting an aliquot of the solution to the HPLC.

Preparation of fungal spores and bacterial cells for infection:

Live cultures: *A. fumigatus* was initially grown on Malt extract agar (MEA) slants for 5–7 days at 37°C. The conidia were collected in sterile PBS with 0.1% Tween 20 (PBST) and passed

through 40µm nylon filters. The conidia were washed twice, counted on a hemocytometer diluted to the desired concentration. The bacterial strains (*E. coli and S. aureus*) were grown in Lysogeny Broth (LB) overnight at 37 ^{o}C with shaking. Next day, the bacteria were sub-cultured and allowed to grow up to the log phase (OD_{600} ~0.8). The cultures were then washed twice and resuspended in PBS to the required concentrations.

Heat-killed cultures: *A. fumigatus* spores were autoclaved at 121°C for 30 minutes to kill all spores. The absence of growth was verified by plating on slants. Both bacterial cultures were incubated at 95°C for 1h to ensure complete heat-killing and lack of growth was verified by growing on LB plates.

PET/CT Imaging

During imaging, the mice were placed on a heating pad to keep them warm throughout scanning. Static PET imaging (10~15 min) was acquired followed by CT for attenuation correction and anatomical coregistration, using Nucline software (Mediso). For all scans, the emission sinograms were corrected for scatter, ^{89}Zr-decay, random, and dead time. The resulting histograms were then reconstructed applying Fourier rebinning and 3D ordered subject expectation maximization algorithm (OSEM-3D).

Grocott's methamine silver nitrate (GMS) and Immunofluorescence Staining

After PET/CT imaging, *A. fumigatus* infected mice were transcardially perfused with normal saline and 4% PFA prior to tissue collection. The lungs or thighs were cryo-sectioned into 10–15 um sections and then stained with GMS (ScyTek Laboratories Inc., West Logan, UT) to identify

fungal hyphae or spores. Images were collected using an Olympus VS200 Slide Scanner (Tokyo, Japan).

For immunofluorescence staining, frozen sections from *A. fumigatus* infected thigh muscle were fixed with ice cold acetone, rinsed with PBS, and blocked with 10% bovine serum albumin for 30 min at room temperature. The slices were incubated with 20ug/mL antibody or fragment for 1 h at room temperature and then visualized with Alexa Fluor 647 conjugated secondary antibodies (EarthOx, Millbrae, CA) under a ZEISS Axio Imager M2 Microscope (Oberkochen, Germany).

Supplementary figures:

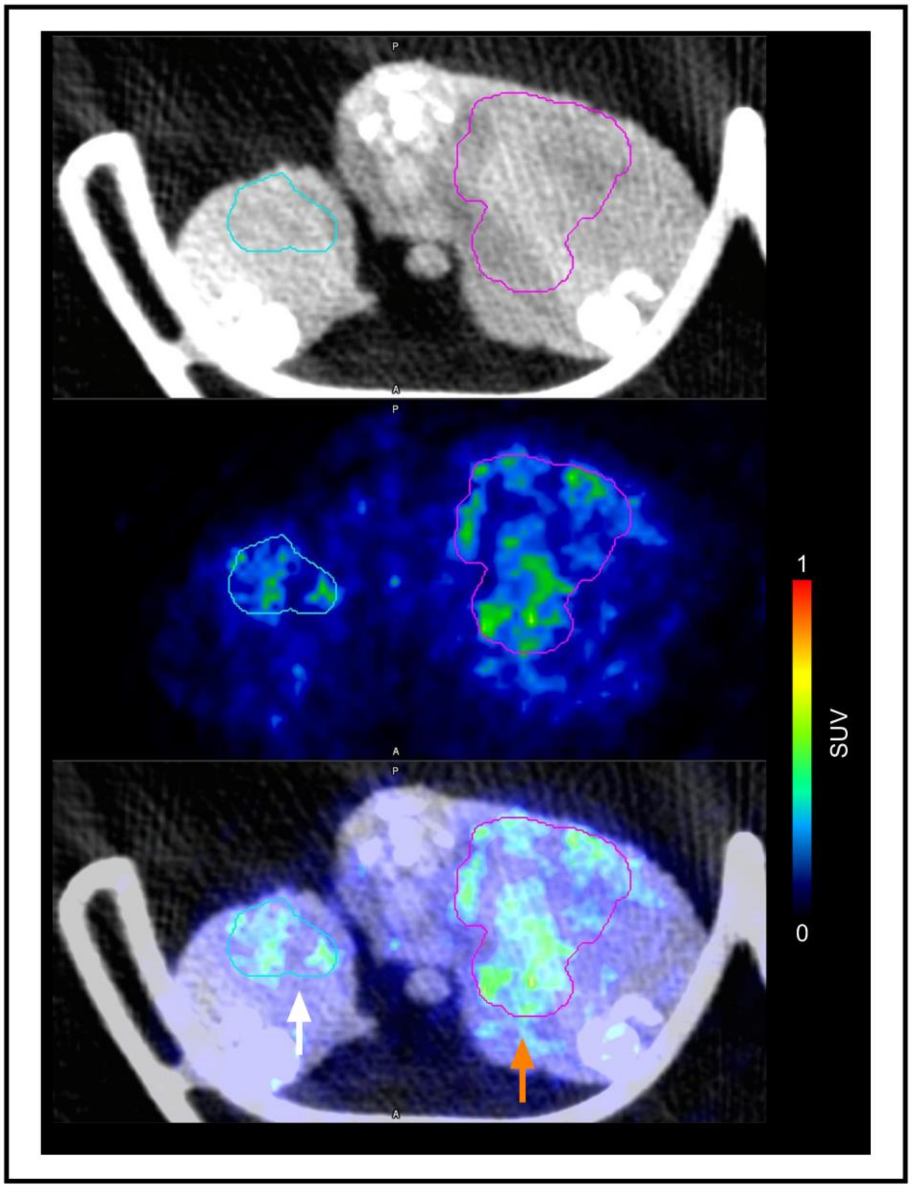

Fig.S1. Representative axial CT (top), PET (middle) and PET/CT overlay (bottom) images showing VOIs drawn for the live (orange arrows) and heat-killed (white arrows) inoculation sites in the *A. fumigatus* myositis mouse after injection of ^{89}Zr-HA-βG-Fab.

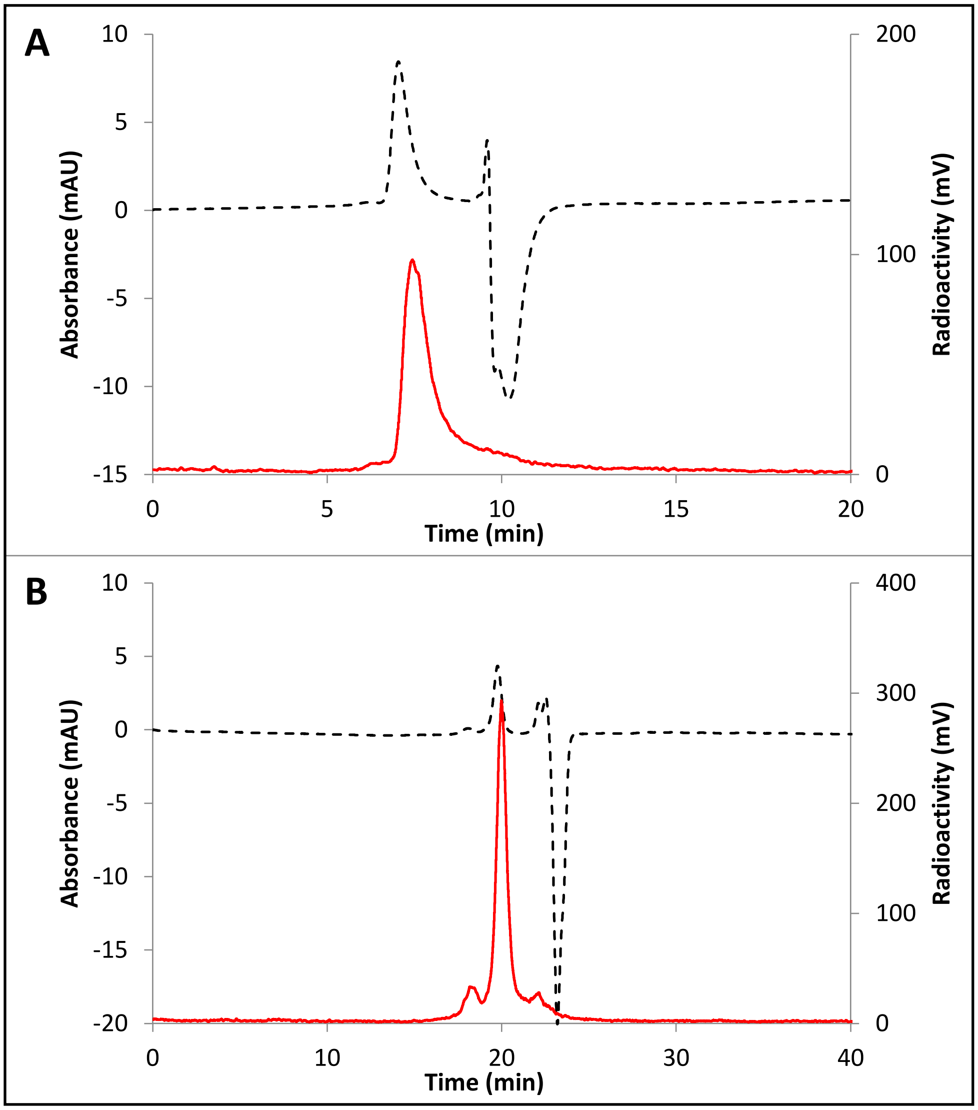

Fig.S2. Representative chromatograms of (A) ^{89}Zr-HA-βG-Ab and (B) ^{89}Zr-HA-βG-Fab. Dotted line indicates the ultraviolet (UV) absorbance and red line indicates the radioactivity.

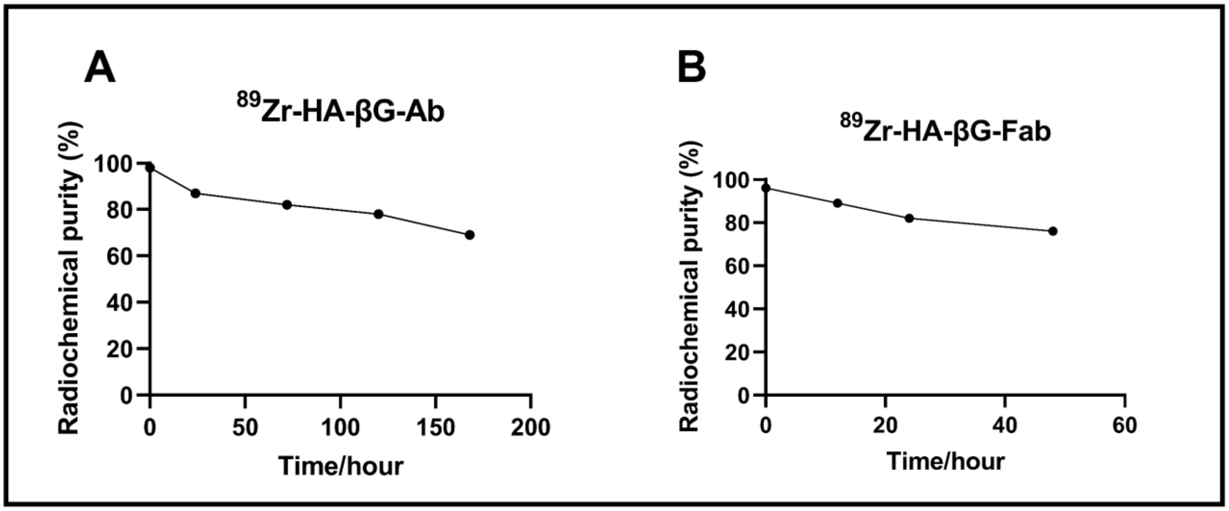

Fig.S3. *In vitro* stabilities of (A) ^{89}Zr-HA-βG-Ab and (B) ^{89}Zr-HA-βG-Fab in mouse serum at 37°C at the indicated timepoints.

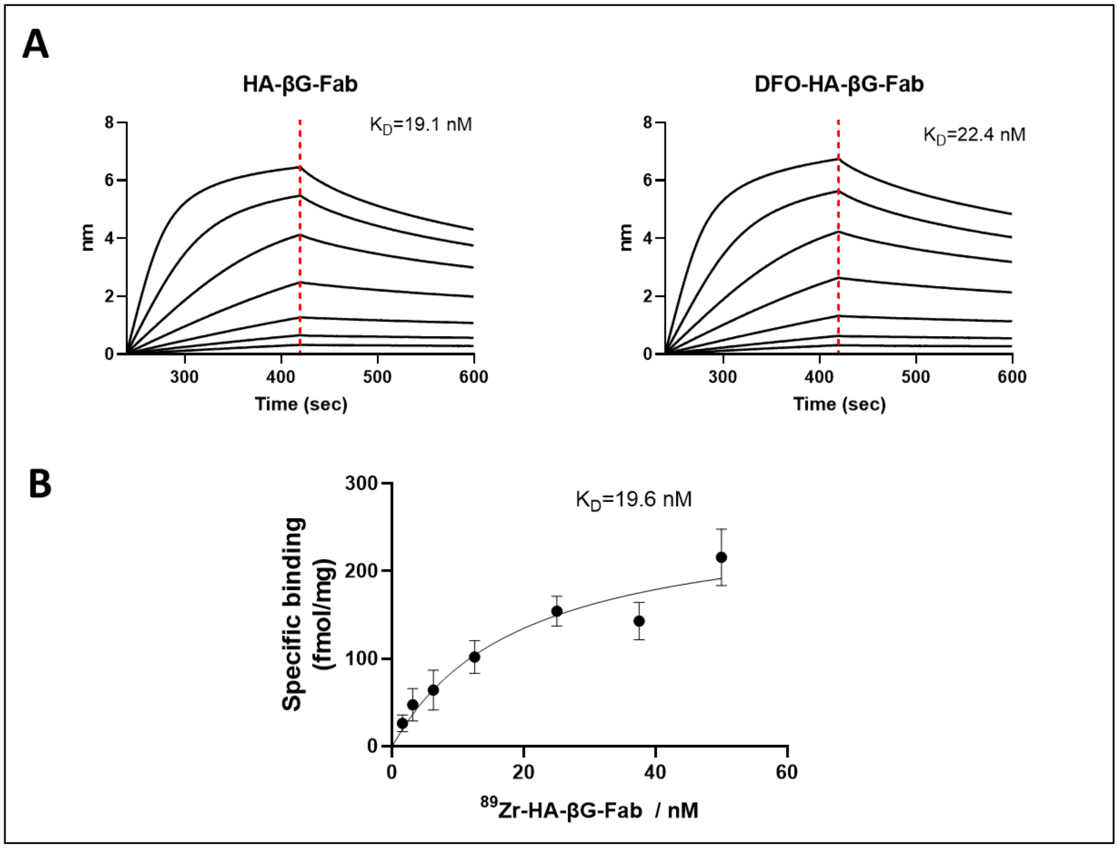

Fig.S4. (A) Binding affinity of HA-βG-Fab and DFO-HA-βG-Fab to biotinylated laminarin was determined by biolayer interferometry. Dissociation constants (K_D) are shown. (B) Saturation binding of ^{89}Zr-HA-βG-Fab using *Candida albicans* cultures.

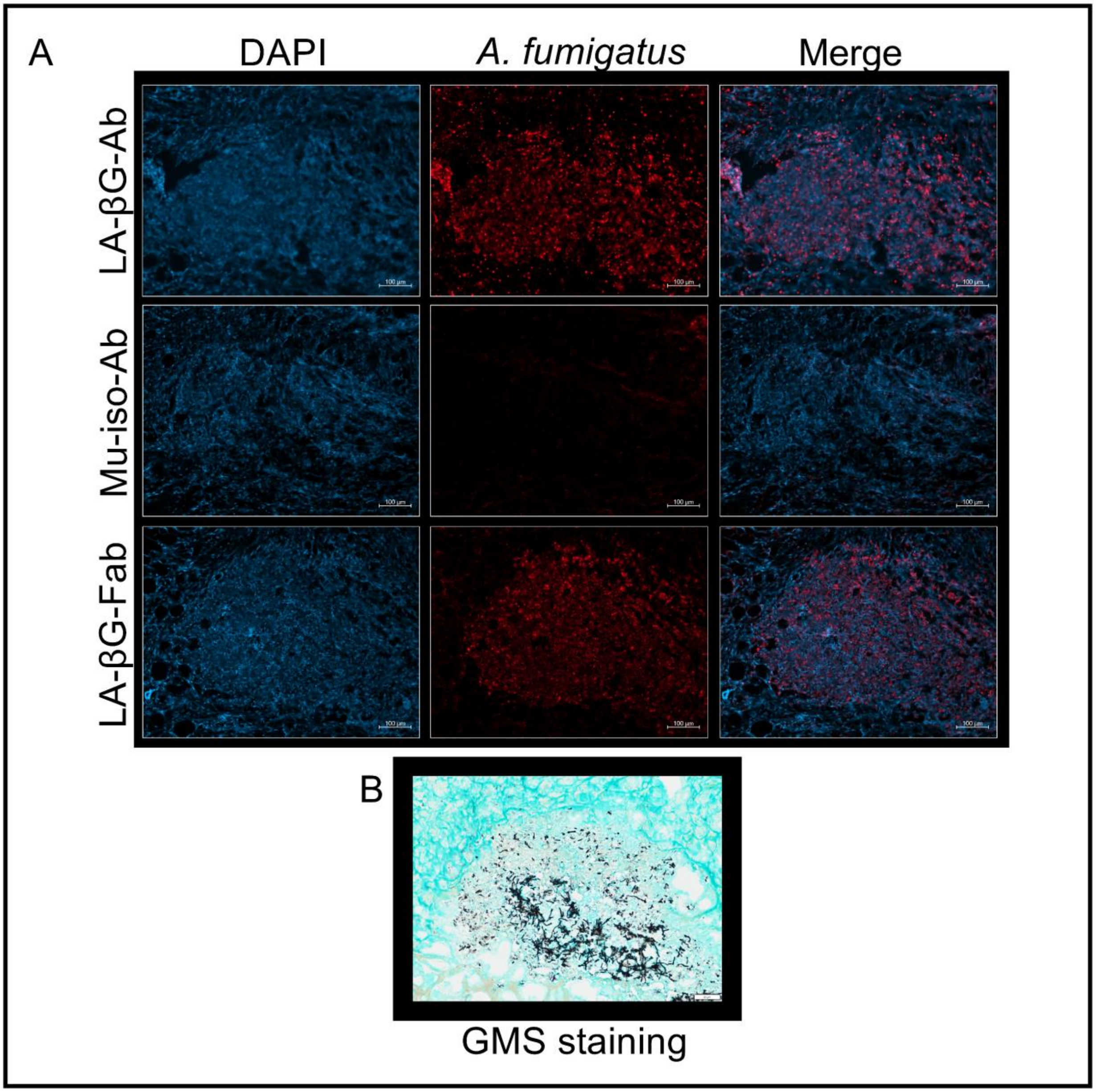

Fig.S5. Validation of LA-βG-Ab and LA-βG-Fab binding to *A. fumigatus* by immunofluorescence microscopy. (A) Immunofluorescence staining of β-glucans in muscle tissues from *A. fumigatus* myositis model, using LA-βG-Ab, Mu-iso-Ab and LA-βG-Fab as primary antibodies/Fabs, Alexa Fluor 647 (Red) labeled as secondary antibody, counterstained with DAPI (blue). All images were acquired under same conditions and displayed at same scale. Scale bar: 100 mm. (B) GMS staining was done on an adjacent slice to confirm fungal infection.

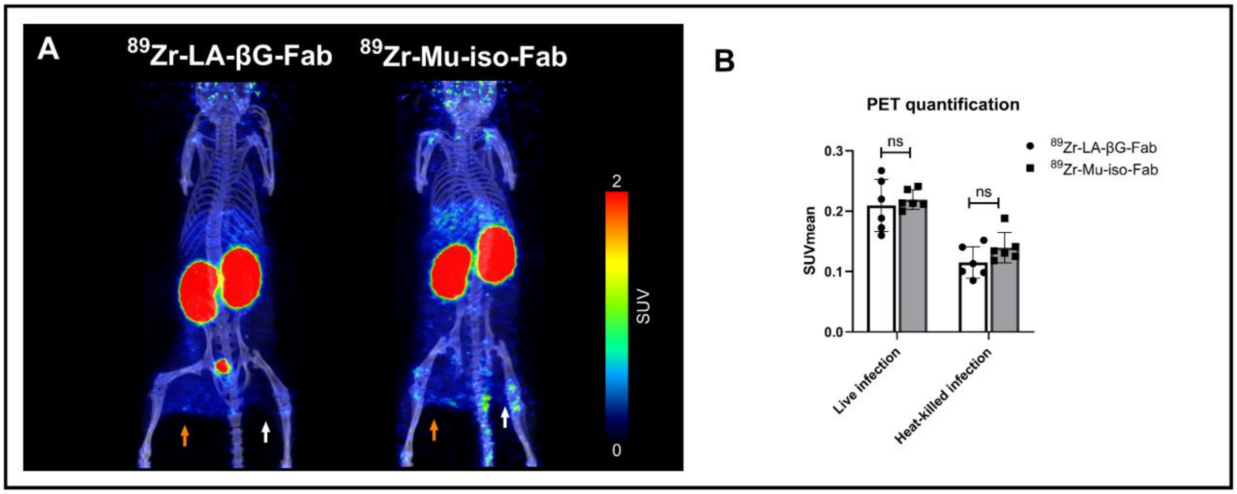

Fig.S6. Static PET/CT imaging of Aspergillus myositis models with ^{89}Zr-LA-βG-Fab and ^{89}Zr-Mu-iso-Fab. (A) Maximum intensity projections (MIPs) of representative PET/CT images at 24 hours post-tracer injection and (B) PET quantification of binding in the live *A. fumigatus* (orange arrows) and heat-killed *A. fumigatus* (white arrows) injection sites (n=6). Unpaired t-test was used for statistical analysis.

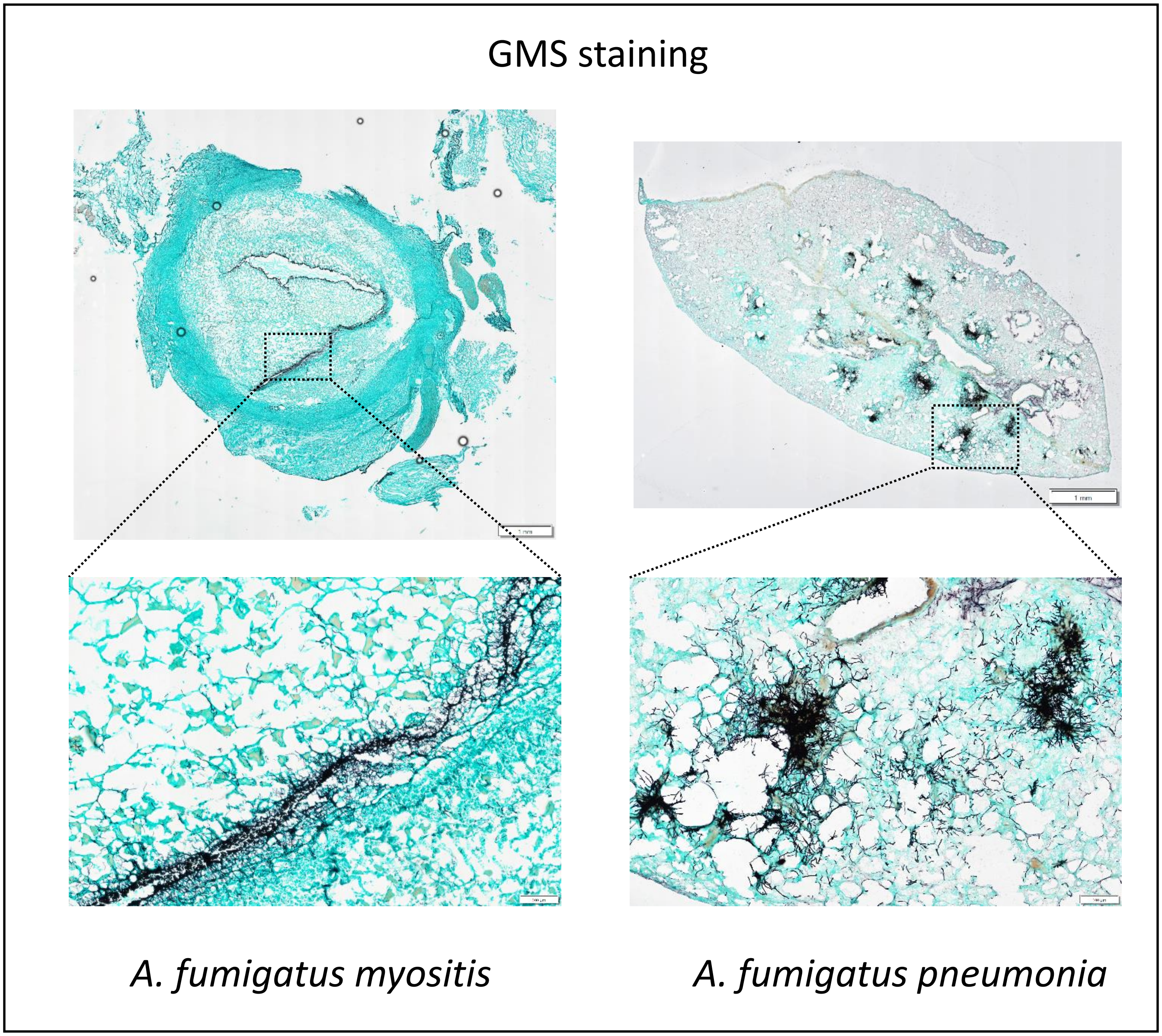

Fig.S7. GMS staining of muscle and lung slices from *A. fumigatus* myositis (A) and pneumonia (B) mouse models. Scale bars, 1 mm (upper panels) and 100 µm (lower panels).

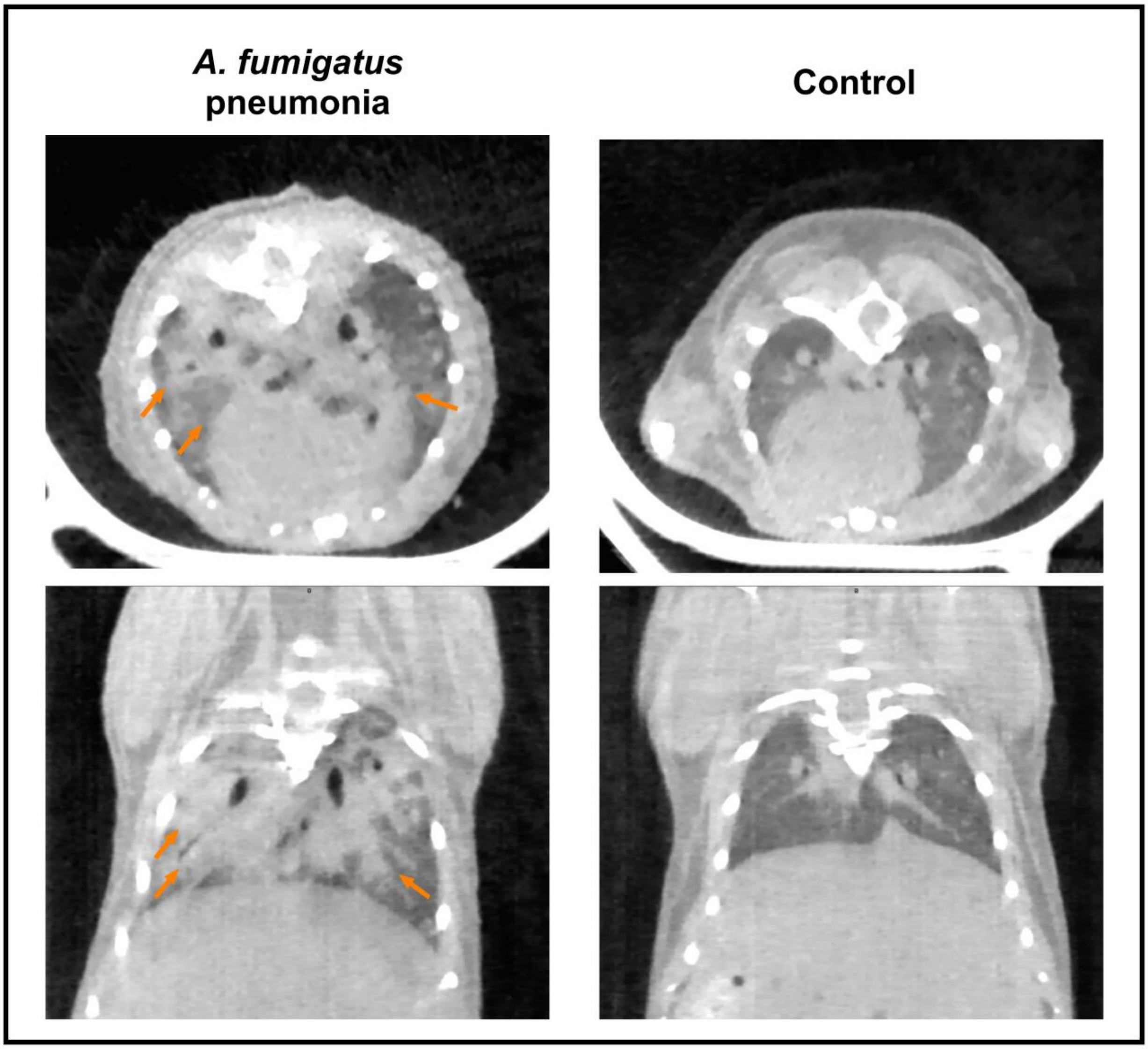

Fig.S8. Representative CT images showing lung infiltrates/consolidation (orange arrow) in the lungs of an *A. fumigatus* pneumonia model compared to a healthy control animal.

Table.S1: Summary of antibodies and antibody fragments used.

	Full antibody	Antibody fragment
Mouse anti-1,3-β-glucan (2G8)	LA-βG-Ab	LA-βG-Fab
Mouse isotype control	Mu-iso-Ab	Mu-iso-Fab
Rabbit anti-β-glucan (B3149M)	HA-βG-Ab	HA-βG-Fab
Rabbit isotype control	Ra-iso-Ab	Ra-iso-Fab

LA: low affinity; HA: high affinity; βG: anti-β-glucan; iso: isotype control; Ab: antibody; Fab: antibody binding fragment; Mu: murine; Ra: Rabbit.
